# Different Characteristics of Aquaporin-4 and Myelin Oligodendrocyte Glycoprotein Antibody-Seropositive Male Optic Neuritis in China

**DOI:** 10.1155/2019/4015075

**Published:** 2019-04-01

**Authors:** Honglu Song, Huanfen Zhou, Mo Yang, Junqing Wang, Hongjuan Liu, Mingming Sun, Quangang Xu, Shihui Wei

**Affiliations:** ^1^Department of Ophthalmology, Chinese PLA General Hospital, Beijing, China; ^2^Department of Ophthalmology, Bethune International Peace Hospital, Shijiazhuang, Hebei, China

## Abstract

**Purpose:**

To describe different clinical characteristics and prognosis of optic neuritis (ON) in male patients with seropositive aquaporin-4 antibody (AQP4-Ab) or myelin oligodendrocyte glycoprotein antibody (MOG-Ab) in China.

**Method:**

Males with ON were recruited from the Neuro-ophthalmology Department of the Chinese People's Liberation Army, General Hospital from January 2016 to February 2018. They were assigned to two groups based on antibodies status: MOG-Ab-seropositive ON (MOG-ON) and aquaporin-4 Ab-seropositive ON (AQP4-ON).

**Results:**

Seventy-six male patients were assessed, including 44 MOG-ON (57.9%) and 32 AQP4-ON (42.1%). The MOG-ON patients were significantly younger at onset compared to the AQP4-ON group (*p* < 0.001). Frequencies of optic disc swelling, presence of abnormal autoimmune antibodies, and elevated levels of CSF IgG were significantly higher in the AQP4-ON group than the MOG-ON group (*p*=0.040, *p*=0.016, and *p*=0.10, respectively). At the final visit, 85.3% of MOG-ON eyes had increased visual acuity (≥0.5) compared to 35.1% of AQP4-ON eyes (*p* < 0.001). The ratio of this steroid-dependent condition is higher in MOG-ON patients than the AQP4-ON group (*p* < 0.001). The ratio of conversion to NMO is higher in the AQP4-ON group than the MOG-ON group, with more AQP4-ON patients developing NMO by the follow-up (*p*=0.012). MOG-ON patients had thicker average peripapillary retinal nerve fiber layers and macular ganglion cell-inner plexiform than AQP4-ON patients (*p*=0.008 and *p*=0.012, respectively). Orbital MRI revealed more AQP4-ON patients had chiasmal involvement than MOG-ON patients (*p* < 0.001).

**Conclusion:**

Male MOG-ON patients had different clinical features including earlier age of onset, higher optic disc swelling ratio, better visual acuity recovery, thicker peripapillary retinal nerve fiber and macular ganglion cell-inner plexiform layers, and less chiasmal involvement than male AQP4-ON patients. Serum antibody may be a potential biomarker for determining visual prognosis in male ON.

## 1. Introduction

Optic neuritis (ON), an inflammatory demyelinating disorder of the optic nerve, is the most common type of optic neuropathy affecting young individuals. Much of our understanding of ON comes from the Optic Neuritis Treatment Trial (ONTT) which demonstrated that the majority (77.2%) of enrolled ON patients were women [1]. ON may occur as an idiopathic isolated event or in conjunction with various central nervous system (CNS) demyelinating diseases, such as multiple sclerosis (MS), neuromyelitis optica spectrum disorder (NMOSD), and acute disseminated encephalomyelitis (ADEM) [2–5]. It is well known that the aquaporin-4 (AQP4) antibody has a crucial role as a marker in the diagnosis and prognosis of ON. Additionally, recent findings have highlighted the potential value of the myelin oligodendrocyte glycoprotein (MOG) antibody to differentiate other ON phenotypes [6, 7].

NMOSD-ON is an inflammatory disease characterized by a high female predominance, but the effect of sex on patients with NMOSD-ON has not been fully evaluated [8, 9]. The etiology and clinical characteristics of male ON are not as clear as those in female patients [10]. However, there have only been a few reports on sex-based differences among NMOSD-ON worldwide [10–13]. The number of male cases in these reports was too small for drawing clear conclusions, and the clinical features of male ON remain unclear. To date, little is known about the frequency of male AQP4-ON and MOG-ON, or the different characteristics in male Chinese patients with ON. Therefore, this cohort study recruited Chinese males with ON, including those with AQP4- and MOG-ON. The clinical characteristics and prognosis of male patients with antibody-seropositive male ON in China were then assessed.

## 2. Methods

### 2.1. Patient Enrollment

Clinical data were retrospectively collected from hospitalized male patients diagnosed with antibody-seropositive ON at the Department of Neuro-ophthalmology in the Chinese People's Liberation Army General Hospital (PLAGH) from January 2016 to February 2018. This study was approved by the Chinese PLAGH Ethics Committee and was conducted following the current Declaration of Helsinki ethical guidelines and applicable Chinese laws. Informed consent was obtained from patients or their guardians. All enrolled patients were treated with intravenous methylprednisolone (dose 20 mg/kg/day for children, 1 g/day for adults) for 3–5 days followed by a taper of oral prednisone (starting dose 1 mg/kg/day) with variable durations, based on the subtype of and recovery from male optic neuritis. Follow-up data were obtained during the return visit clinical examinations and follow-up surveys over the telephone with the patients or their guardians. All patients were followed for at least 6 months.

### 2.2. Diagnostic Criteria

ON was diagnosed in accordance with ONTT guidelines [1]. The detailed inclusion criteria were the following: (1) the male patients presented with acute loss of visual acuity or visual field, with or without eye pain during their ON episode and (2) at least one of the following objective evidences of visual abnormalities: relative afferent pupillary defect, abnormal visual evoked potential, and visual field defect.

Exclusion criteria included the following: (1) any other types of optic neuropathy, including, compressive, vascular, toxic, metabolic, infiltrative, or hereditary optic neuropathy, (2) the presence of craniocerebral lesions other than those from demyelinating diseases involving the optic chiasm or optic pathway downstream of the optic chiasm and optic cortex, (3) the presence of ametropia, glaucoma, anterior segment, or retinal or macular diseases, and (4) unknown serum MOG and AQP4 antibodies status.

### 2.3. Laboratory Examinations

Serum and cerebrospinal fluid (CSF) samples were obtained from enrolled male patients within 1-month post ON attack. Serum samples were tested for the presence of AQP4 and MOG antibodies using a cell-based assay (Euroimmun, Lübeck, Germany). Based on their serum antibody status, the enrolled male patients were categorized as either MOG-ON or AQP4-ON.

All patient sera were tested for auto-antibodies, including antinuclear antibody (ANA), human leukocyte antigen-B27 (HLA-B27), antithyroglobulin antibodies (ATAs), antithyroid peroxidase autoantibody (anti-TPOAb), anti-Sjögren's syndrome-related antigen A (SSA), anti-Sjögren's syndrome-related antigen B (SSB), anticardiolipin antibodies (ACLs and b2-GPI), antineutrophil cytoplasmic antibody (ANCA), rheumatoid factor (RF), and antiperinuclear factor (APF) in the Examination Center for Biomedical Research of PLAGH. The CSF was tested for white blood cell count and protein and IgG levels. Additionally, the criteria of elevation in CSF tests were defined as white cell count elevated (white cell count >10/*µ*l), protein elevated (protein >400 mg/L), and IgG level elevated (IgG level >3.4 mg/dL).

### 2.4. Neuro-Ophthalmology Examinations

Best-corrected visual acuity (BCVA) as the main outcome was examined by the standard table of vision logarithms at 5 m. Those unable to read any letters at 1 m were further examined by finger counts, hand movements, or perceiving light. A good visual outcome was defined as a final BCVA of 0.5 or better, and a very poor outcome was defined as a BCVA of <0.1. BCVAs were transformed into logarithm of the minimum angle of resolution (logMAR) values. Among these values, counting the number of ﬁngers held before the eye was transformed as a logMAR value of 1.85, hand movement as a logMAR value of 2, light perception as a logMAR value of 2.7, and no light perception as a logMAR value of 3.0 [14]. Peripapillary retinal nerve fiber layers (pRNFLs) and macular ganglion cell-inner plexiform layers (mGCIPLs) were assessed at least 3 months after ON attack using high-definition spectral domain optical coherence tomography (SD-OCT: Carl Zeiss Meditec, Dublin, CA, USA).

All enrolled patients underwent orbital magnetic resonance imaging (MRI) with T2-weighted image and gadolinium-enhanced T1 sequences. The anterior visual pathways were divided into five segments: orbital, canalicular, intracranial, the optic chiasm, and tract [15]. Head or spinal MRIs were performed in these patients with myelitis or systemic symptoms.

### 2.5. Statistical Analysis

Demographic parameters were described and compared between these two different patient cohorts. Statistical analyses were conducted using SPSS 20.0 software (IBM Corporation, New York, USA). Student's *t*-test was used for parametric comparisons while the Mann–Whitney *U* test was used for nonparametric comparisons. Categorical data were analyzed using Pearson *χ*^2^ or Fisher's exact test where appropriate. All probability values were two-tailed and considered to be significant at *p* < 0.05.

## 3. Results

### 3.1. Demographics and Clinical Characteristics

Table 1 summarizes the demographic and clinical characteristics of the 76 (132 eyes) male patients enrolled in the present study. The mean age at onset was 27.11 ± 16.74 years (range of 3–72 years). Nineteen patients (25.0%) experienced bilateral involvement while 57 (75.0%) experienced unilateral involvement. The follow-up duration ranged from 6 to 158 months with a mean time of 38.67 ± 38.78 months. 283 female patients with ON were seen during the same period. All enrolled male patients underwent serum MOG-Ab and AQP4-Ab testing, which revealed 32 patients had AQP4-ON (42.1%) and 44 had MOG-ON (57.9%). No patients were found to be both MOG-Ab and AQP4-Ab seropositive. In this study cohort, 77 eyes (81.1%) were scored at 0.1 or worse at the initial attack, and 84 eyes (63.6%) were 0.5 or better at the final visit. Forty-four patients (57.9%) received immunosuppressant therapy, which included azathioprine (6, 7.9%), mycophenolate (17, 22.4%), and rituximab (21, 27.6%).

Table 2 presents the demographic and clinical characteristics of the different male ON subgroups. In the AQP4-ON group, the mean age at onset was 36.4 years (range of 12–72 years) compared to 20.3 years (range of 3–61 years) for the MOG-ON group. MOG-ON patients were significantly younger than AQP4-ON patients (*p* < 0.001). The MOG-ON cohort had a higher ratio of pediatric patients (<18 years) than the AQP4-ON cohort (59.1% vs. 9.4%, *p* < 0.001). The frequencies of bilateralism at first onset and ocular pain were not significantly different among the two groups. The frequencies of optic disc swelling and presence of abnormal autoimmune antibodies were significantly higher in the AQP4-ON group than the MOG-ON group (*p*=0.040 and *p*=0.016). The levels of protein and IgG in the CSF were significantly higher in AQP4-ON patients than MOG-ON patients (*p*=0.024 and *p*=0.003). The ratio of elevated levels of IgG was also significantly higher in the AQP4-ON group than the MOG-ON group (*p*=0.010).

### 3.2. Visual Outcomes and Clinical Prognosis

Table 3 compares the visual outcomes and clinical prognosis of male in the MOG-ON and AQP4-ON groups. After treatment of the initial attack, 53 eyes (93.0%) in the MOG-ON group had good visual recovery (≥0.5) compared to eyes in the AQP4-ON group (18, 47.4%) (*p* < 0.001). At the final visit, 64 eyes (85.3%) had a better visual recovery (≥0.5) in the MOG-ON group compared to the AQP4-ON group (20, 35.1%) (*p* < 0.001). The comparison of logMAR recovery after treatment after initial attack or at the final visit revealed a significant difference between AQP4-ON and MOG-ON male patients (*p* < 0.001 and *p* < 0.001, respectively). During the follow-up of the cohorts, 53 patients (69.7%) experienced at least one episode of recurrence of ON. MOG-ON patients had a higher recurrence rate than the AQP4-ON patients (81.2% vs. 61.4%, respectively, *p*=0.062). Notably, 22.4% (17/76) of male ON patients were steroid dependent and tended to relapse once the steroid dose was reduced significantly (5–20 mg daily) or stopped within 30 days; the ratio of this condition is higher in MOG-ON patients than AQP4-ON patients (59.3% vs. 3.8%, *p* < 0.001). The ratio of conversion to NMO over the course of the follow-up is higher in the AQP4-ON group than the MOG-ON group (28.1 vs. 6.8%, respectively, *p*=0.012). Two children in the MOG-ON group had an ON attack concurrently with an episode of ADEM and received a final diagnosis of ADEM-ON.

### 3.3. OCT Measurements

Table 4 presents the pRNFL and mGCIPL thicknesses measured by spectral domain-OCT. The pRNFL thickness measurements were performed on 44 AQP4-ON and 49 MOG-ON eyes. The MOG-ON eyes were found to have less loss of pRNFL than the AQP4-ON group (*p*=0.008). The mGCIPL thicknesses measurements were performed on 37 AQP4-ON and 49 MOG-ON eyes. There was less reduction in the average mGCIPL thickness in the MOG-ON group than the AQP4-ON group (58.63 ± 6.75 vs. 54.51 ± 8.10 *μ*m, respectively, *p*=0.012).

### 3.4. MRI Manifestation

Table 5 presents the optic nerve MRI results, which revealed T2 hyperintensity with or without enhancement. No significant differences in the rate of intraorbital of optic nerve T2 lesions were present in the AQP4-ON group compared with the MOG-ON group (94.4% vs. 95.7%, *p* > 0.999). The AQP4-ON and MOG-ON groups had almost the same incidence of intracanalicular involvement in T2-weighted images (75.9% vs. 67.1%, *p*=0.285). The intracranial portion of the optic nerve was involved more in patients with AQP4-ON than MOG-ON (59.3% vs. 42.9%, *p*=0.070). The proportion of patients experiencing chiasmal involvement was higher in the AQP4-ON cohort than the MOG-ON cohort (48.4 vs. 11.6%, respectively, *p* < 0.001). Furthermore, only three patients (9.7%) with AQP4-ON displayed optic tract involvement.

## 4. Discussion

In this single-center study, we preferentially evaluated the clinical features and prognosis according to AQP4-Ab and MOG-Ab serostatus in Chinese males with ON. Sex may influence the clinical characteristics, disease course, and severity in ON. Previous studies showed female predominance in ON, especially in NMOSD-ON patients [8]. Our previous study [16] showed that atypical ON with seronegative AQP4-Ab and MOG-Ab had distinct clinical features which included a male predominance, low relapse rate, and worse visual prognosis, which highlighted the need to increase our understanding of sex-specific aspect of ON to better prognostic assessment and further insight into ON pathophysiology. We evaluated our cohort's Chinese male patients with antibody-seropositive ON (Ab-ON) and found 57.9% (44/76) of these patients were seropositive for MOG-Ab. Sun et al. [13]. tested the serum of 97 Chinese patients with NMOSD, 15 (15.5%) of which were male patients, with 46.2% of these male patients found to be AQP4-Ab-positive; however, MOG-Abs were not tested for in that study. To the best of our knowledge, our present study which focused on male patients and expanded knowledge of clinical characteristics and prognosis between male MOG-ON and male AQP4-ON was the first of its kind in China.

In our cohort of male ON patients, we found MOG-ON patients were significantly younger at onset compared to AQP4-ON patients. The MOG-ON cohort had a higher ratio of pediatric patients (<18 years) than the AQP4-ON group (59.1% vs. 9.4%, *p* < 0.001). Zhao et al. [18] reported similar results in ON patients of all ages, finding that the onset in MOG-ON occurs at a younger age than AQP4-ON (range: 5–63 years vs. 8–72 years, respectively). The frequencies of optic disc swelling, presence of abnormal autoimmune antibodies, and elevated levels of CSF IgG were significantly higher in the AQP4-ON group than the MOG-ON group (*p*=0.040, *p*=0.016, and *p*=0.10). Previous studies had shown that coexisting autoimmunity was rare in MOG-ON patients, whereas coexisting autoimmune disorders were present in more than one-third of AQP4-ON patients [19].

In the final visit, 85.3% of the eyes in male MOG-ON patients had better recovery of visual acuity (VA) than male AQP4-ON patients (35.1%), which is consistent with other studies on Asian ON which included all populations [20, 21]. In the male MOG-ON subgroup, 59.3% (16/27) of relapsed ON patients had a clinical course consistent with chronic relapsing inflammatory optic neuropathy (CRION) [22]. Relapses occurred upon steroid withdrawal or dose reductions, which coincided with Ramanathan et al.'s findings that MOG-ON patients are highly responsive to steroids, but vulnerable to relapse upon steroid reduction and cessation [23].

MOG-Abs can be detected in NMOSD with AQP4-Ab seronegative, recurrent optic neuritis, transverse myelitis, multiphasic acute disseminated encephalomyelitis, and combined central and peripheral demyelination (CCPD) syndromes; [24–26] a proportion of these patients will develop a relapsing type of disease [23, 27]. In our study, 61.4% of the male patients in the MOG-ON cohort presented with the relapsing form of the disease. ADEM followed by ON is a rare, but a distinct clinical phenotype in children [3, 28]. In our cohort, two children in the MOG-ON group had an ON attack during an episode of ADEM. However, due to the low number of ADEM cases, we were unable to identify a relationship between the titer and severity of ADEM-ON. The ratio of conversion to NMO during the time until the last follow-up is higher in the AQP4-ON group than the MOG-ON group (28.1 vs. 6.8%, respectively, *p*=0.012).

OCT measurements of the pRNFL and mGCIPL thickness are currently extensively used as structural markers for axonal loss in ON patients and to differentiate between ON subtype [29, 30]. Our study found significant differences between the AQP4-ON and MOG-ON groups in terms of OCT measurements. The average pRNFL and mGCIPL were preferentially damaged in AQP4-ON eyes compared to the MOG-ON eyes, which is consistent with previous studies in ON with all populations [21, 31, 32]. Pache et al.'s [33] study had shown that no significant differences in pRNFL and mGCIPL thickness between MOG-ON and AQP4-ON patients. Havla et al. [34] yielded controversial results in NMOSD patients, finding that MOG-Ab-positive NMOSD had more retinal atrophy than AQP4-Ab-positive NMOSD patients. The mechanism of this paradoxical result is unknown. We presumed that it was related to differences in the preferred populations used for the studies.

In our cohort, optic nerve orbital segment involvement occurred in 95.2% of male patients with ON, whereas the optic chiasm and optic tract lesion were involved in more AQP4-ON patients than MOG-ON patients. This indicated AQP4-ON lesions were more likely to involve the posterior optic nerve segment, which is consistent with previous studies in ON including all populations [35, 36].

This study had several limitations. First, the nature of the retrospective study from a single center introduces selection bias, precluding the confirmation of the cause and effect relationship. Second, AQP4-Ab or MOG-Ab titers were not all measured within 1 month after ON onset, so the relationship between titer and prognosis was not discerned. At last, the follow-up was not standardized and relatively short. Therefore, prospective studies with a larger sample size and involving male patients of varied ethnicities are required to clarify the influence of MOG-Ab and AQP4-Ab on the final prognosis of male ON patients.

In conclusion, based on the results of the current study, male ON is quite common in Chinese demyelinating patients. Male MOG-ON patients had different clinical features including earlier age of onset, higher optic disc swelling ratio, better recovery of visual acuity, thicker peripapillary retinal nerve fiber and macular ganglion cell-inner plexiform layers, and less chiasmal involvement than male AQP4-ON patients. Serum antibody may be a potential biomarker for determining visual prognosis in male ON.

## Figures and Tables

**Table 1:** Demographic and clinical characteristics of the male Ab-ON' cohort.

	Total	Percentage
Number of patients (eyes)	76 (132)	
Age of onset, year		
Mean	27.11 ± 16.74	
Range	3–72	
Follow-up, month		
Mean ± SD	38.67 ± 38.78	
Range	6–158	
Experience		
Bilateral attack	19	25.0
Unilateral attack	57	75.0
Serum antibodies status		
Positive AQP4-Ab	32	42.1
Positive MOG-Ab	44	57.9
Optic disc edema	22	28.9
Ocular pain	47	61.8
Abnormal autoimmune antibody	18	23.7
Presenting BCVA		
≤0.1	40	30.3
0.1–0.5	8	6.1
≥0.5	84	63.6
Treatment		
IV methylprednisolone	73	96.1

AQP4, aquaporin-4; MOG, myelin oligodendrocyte glycoprotein; IV, intravenous; BCVA, best-corrected visual acuity.

**Table 2:** Demographic and clinical characteristics in male patients with AQP4- and MOG-ON.

	AQP4-ON	MOG-ON	*p* value
No. of patients, *n* (%)	32 (42.1)	44 (57.9)	
No. of eyes	57	75	
Mean age at onset (range)	36.4 (12–72)	20.3 (3–61)	<0.001^*∗∗*^
Age at first attack <18 years (*n*, %)	3 (9.4)	26 (59.1)	<0.001^*∗∗*^
Bilaterality at first onset, *n* (%)	6 (18.8)	13 (29.5)	0.283
Ocular pain, *n* (%)	17 (53.1)	30 (68.2)	0.182
Optic disc swelling, *n* (%)	5 (15.6)	17 (38.6)	0.040^*∗*^
Abnormal autoimmune antibody, *n* (%)	12 (37.5)	6 (13.6)	0.016^*∗*^
ANA	8 (25.0)	0 (0.0)	
HLA-B27	1 (3.1)	2 (4.5)	
ATAs/anti-TPOAb	4 (12.5)	2 (4.5)	
Anti-SSA/anti-SSB	0 (0.0)	0 (0.0)	
ACL/*β*2-GPI	3 (9.4)	2 (4.5)	
ANCA	0 (0.0)	0 (0.0)	
RF	0 (0.0)	0 (0.0)	
APF	1 (3.1)	0 (0.0)	
No. of lumbar punctures	27	37	
CSF white cell count, mean ± SD (n/mm^3^)	2.81 ± 3.16	6.32 ± 20.14	0.374
White cell counts elevated	1 (3.7)	4 (10.8)	0.387
CSF protein, mean ± SD (g/L)	437.3 ± 141.9	360.8 ± 119.9	0.024^*∗*^
Protein elevated	15 (57.7)	15 (40.5)	0.180
CSF IgG level, mean ± SD (mg/dL)	3.46 ± 1.54	2.43 ± 1.15	0.003^*∗∗*^
IgG level elevated	12 (46.2)	6 (16.2)	0.010^*∗*^

^*∗*^ *p* < 0.05; ^*∗∗*^*p* < 0.01; ANA, antinuclear antibody; HLA-B27, human leukocyte antigen-B27; ATAs, antithyroglobulin antibodies; anti-TPOAb, antithyroid peroxidase autoantibody; anti-SSA and SSB, anti-Sjögren's syndrome A and B; ACL, anticardiolipin antibody; *β*2-GPI, anti-*β*2-glycoprotein I; ANCA, antineutrophil cytoplasmic antibodies; RF, rheumatoid factor; APF, antiperinuclear factor; CSF, cerebrospinal fluid; ON, optic neuritis.

**Table 3:** Comparison of visual outcomes and clinical prognosis in male AQP4-ON and MOG-ON groups.

	AQP4-ON	MOG-ON	*p* value
No. of patients	32	44	
BCVA at nadir at first onset, eyes, *n*	38	57	0.915
≤0.1	31 (81.6)	46 (80.7)	
0.1–0.5	6 (15.8)	8 (14.0)	
≥0.5	1 (2.6)	3 (5.3)	
BCVA recovery at first onset, eyes, *n*	38	57	<0.001^*∗∗*^
≤0.1	16 (42.1)	4 (7.0)	
0.1–0.5	4 (10.5)	0 (0.0)	
≥0.5	18 (47.4)	53 (93.0)	
BCVA recovery at last follow-up, eyes, *n*	57	75	<0.001^*∗∗*^
≤0.1	32 (56.1)	8 (10.7)	
0.1–0.5	5 (8.8)	3 (4.0)	
≥0.5	20 (35.1)	64 (85.3)	
LogMAR at nadir at first onset, mean ± SD	1.92 ± 0.98	1.70 ± 0.88	0.255
LogMAR recovery at first onset, mean ± SD	1.05 ± 1.14	0.19 ± 0.59	<0.001^*∗∗*^
LogMAR recovery at last follow-up, mean ± SD	1.24 ± 1.10	0.26 ± 0.62	<0.001^*∗∗*^
Follow-up, month, mean ± SD	40.72 ± 39.11	37.18 ± 38.93	0.697
Recurrent ON in follow-up, *n* (%)	26 (81.2)	27 (61.4)	0.062
Relapse after steroid reduction or cessation, *n* (%)	1 (3.8)	16 (59.3)	<0.001^*∗∗*^
Time of first to second attack (months)	19.35 ± 26.52	24.18 ± 36.93	0.593
Relapse times in follow-up, mean ± SD	1.78 ± 1.52	2.18 ± 3.12	0.462
ARR, mean ± SD	0.73 ± 0.82	0.68 ± 1.06	0.820
Conversion to NMO, *n* (%)	9 (28.1)	3 (6.8)	0.012^*∗*^

^*∗*^ *p* < 0.05; ^*∗∗*^*p* < 0.01; ON, optic neuritis; BCVA, best-corrected visual acuity; ARR, annualised relapse rate; NMO, neuromyelitis optica; logMAR, logarithm of the minimum angle of resolution.

**Table 4:** Comparison of pRNFL and macular GCIPL thickness in male AQP4-ON and MOG-ON.

	AQP4-ON	MOG-ON	*P* value
pRNFL (*μ*m), eyes (*n*)	44	49	0.008^*∗∗*^
Average thickness (mean ± SD)	61.48 ± 9.79	68.12 ± 13.75	
GCIPL (*μ*m), eyes (*n*)	37	49	0.012^*∗*^
Average thickness (mean ± SD)	54.51 ± 8.10	58.63 ± 6.75	

^*∗*^ *p* < 0.05; ^*∗∗*^*p* < 0.01; pRNFL, peripapillary retinal nerve fiber layer; GCIPL, ganglion cell-inner plexiform layer.

**Table 5:** Comparison of the optic nerve MRIs in AQP4-ON and MOG-ON groups.

	AQP4-ON	MOG-ON	*p* value
No. of eyes	54	70	
Intraorbital, *n* (%)	51 (94.4)	67 (95.7)	>0.999
Intracanalicular, *n* (%)	41 (75.9)	47 (67.1)	0.285
Intracranial, *n* (%)	32 (59.3)	30 (42.9)	0.070
Chiasmal, *n* (%)	15 (48.4)	5 (11.6)	<0.001^*∗∗*^
Tract, *n* (%)	3 (9.7)	0 (0)	0.072

^*∗*^ *p* < 0.05; ^*∗∗*^*p* < 0.01; MRI, magnetic resonance imaging.

## Data Availability

The data used to support the findings of this study are available from the corresponding author upon request.
